# Socio-economic differences in diet, physical activity and leisure-time screen use among Scottish children in 2006 and 2010: are we closing the gap?

**DOI:** 10.1017/S1368980016002949

**Published:** 2017-02-20

**Authors:** Geraldine McNeill, Lindsey F Masson, Jennie I Macdiarmid, Leone CA Craig, Wendy J Wills, Catherine Bromley

**Affiliations:** 1 Rowett Institute of Nutrition and Health, University of Aberdeen, Foresterhill, Aberdeen AB25 2ZD, UK; 2 Public Health Nutrition Group, Institute of Applied Health Sciences, University of Aberdeen, Foresterhill, Aberdeen AB25 2ZD, UK; 3 School of Pharmacy and Life Sciences, Robert Gordon University, Aberdeen, UK; 4 Centre for Research in Primary and Community Care, University of Hertfordshire, Hatfield, UK; 5 ScotCen Social Research, Edinburgh, UK; 6 Scottish Collaboration for Public Health Research and Policy, University of Edinburgh, Edinburgh, UK

**Keywords:** Children, Diet, Activity, Socio-economic status, Scotland

## Abstract

**Objective:**

To investigate socio-economic differences in children’s diet, activity and inactivity and changes in these differences over 4 years during which new policies on food in schools were introduced.

**Design:**

Two cross-sectional surveys in which diet was assessed by FFQ and physical activity and inactivity were assessed by interviewer-administered questionnaire. Socio-economic status was assessed by the area-based Scottish Index of Multiple Deprivation.

**Setting:**

Scotland, 2006 and 2010.

**Subjects:**

Children aged 3–17 years (*n* 1700 in 2006, *n* 1906 in 2010).

**Results:**

In both surveys there were significant linear associations between socio-economic deprivation and intakes of energy, non-milk extrinsic sugars (NMES) as a percentage of food energy, sugar-sweetened beverages, confectionery, crisps and savoury snacks and leisure-time screen use (all higher among children in more deprived areas), while intakes of fruit, fruit juice and vegetables showed the opposite trend. In 2010 children in more deprived areas engaged in more physical activity out of school than those in more affluent areas, but between 2006 and 2010 there was an overall reduction in physical activity out of school. There were also small but statistically significant overall reductions in intakes of confectionery, crisps and savoury snacks, energy and NMES and saturated fat as a percentage of food energy, but no statistically significant change in socio-economic gradients in diet or activity between the two surveys.

**Conclusions:**

Interventions to improve diet and physical activity in children in Scotland need to be designed so as to be effective in all socio-economic groups.

The rise in prevalence of childhood obesity in the 1990s appears to have slowed or reversed in a number of developed countries during the first decade of the 21st century^(^ ^1^ ^–^ ^3^ ^)^. Data from the Health Survey for England show a rise in the prevalence of obesity in 2–15-year-olds from 11 % in 1995 to 19 % in 2004, followed by a plateau at 15–17 % from 2006 to 2014^(^ ^4^ ^)^. The Scottish Health Surveys also show that the prevalence of childhood obesity remained in the range of 16–17 % between 2003 and 2014^(^ ^5^ ^)^ and a stabilising prevalence has been observed in surveys in primary care in England and at school entry in Scotland^(^ ^6^ ^,^ ^7^ ^)^. While the overall prevalence is high in all socio-economic groups, children in more socio-economically deprived households have a higher prevalence of obesity^(^ ^8^ ^,^ ^9^ ^)^ and several studies suggest that there has been a widening of this gap in recent years^(^ ^10^ ^–^ ^12^ ^)^. To address the challenge of childhood obesity a range of policy initiatives have been implemented in both England and Scotland. These include improving the nutritional quality of school meals and restricting the availability of energy-dense foods and snacks within and around schools^(^ ^13^ ^–^ ^15^ ^)^, increasing physical activity in schools, and improving family lifestyle through campaigns such as Change for Life in England and Take Life On in Scotland^(^ ^16^ ^,^ ^17^ ^)^. More recently a UK-wide tax on sugar-sweetened soft drinks has been announced^(^ ^18^ ^)^ with the aim of reducing the intake of ‘free sugars’ (i.e. all sugars added in the preparation of foods as well as those in honey, syrups, fruit juice and fruit juice concentrate), which international and UK advisory committees now suggest should account for about 5 % of total energy intake^(^ ^19^ ^,^ ^20^ ^)^. This target is much lower than estimates of 14–15 % for the very similar category of non-milk extrinsic sugars (NMES)* among 4–18-year-olds in the UK in 2008–2012^(^ ^21^ ^)^. Foods and drinks high in free sugars or NMES are appealing to children and young people, who have a strong preference for sweet tastes^(^ ^22^ ^)^; these foods and drinks are also heavily marketed^(^ ^23^ ^)^ and have a low cost per unit of energy^(^ ^24^ ^)^. High intakes of free sugars or NMES in children increase the risk of dental decay^(^ ^25^ ^)^, may elevate risk factors for cardiometabolic disease^(^ ^26^ ^)^ and could contribute to obesity risk via increased energy intake.

In recent years an association between sedentary behaviour and obesity risk in children has highlighted^(^ ^27^ ^)^, while levels of physical activity give further cause for concern. Between 2008 and 2012 the proportion of 5–15-year-olds in England meeting the recommended level of at least 60 min of moderate-to-vigorous physical activity on each of the last 7 d dropped from 28 to 21 % in boys and from 19 to 16 % in girls^(^ ^4^ ^)^. In Scotland different questions are used; these suggested that the proportion of children aged 3–16 years doing 60 min or more of any activity on each of the last 7 d rose slightly from 71 % in 2009 to 75 % in 2014^(^ ^5^ ^)^.

Children’s diet, in particular their NMES intake, physical activity, inactivity and associations between these variables and socio-economic status, were a particular focus of two cross-sectional surveys which we and others carried out in 2006 and 2010^(^ ^28^ ^,^ ^29^ ^)^. In the current paper we describe the changes between the two surveys in diet, physical activity and inactivity, and overweight and obesity prevalence, and investigate whether there was a change in the socio-economic gradients in these variables.

## Methods

The surveys were carried out in May–September 2006 and June–November 2010 using methods which are described in full elsewhere^(^ ^28^ ^,^ ^29^ ^)^. In brief, samples of children aged 3–16 years in eighty postcode sectors in Scotland in 2006 and 127 sectors in 2010 were drawn from Her Majesty’s Revenue and Customs’ Child Benefit Register (a national register used for child welfare payments which, at the time of the surveys, was awarded to all parents/guardians irrespective of income). After an initial opt-out period the parents/guardians were sent a semi-quantitative FFQ. The FFQ was chosen rather than a 24 h recall as there was no automated 24 h recall available for children in Scotland in 2006 and the FFQ is a more cost-effective instrument for large surveys than interviewer-administered 24 h recalls. The FFQ was adapted for children from one widely used for adults in Scotland^(^ ^30^ ^)^, with a validation study of an earlier version in 121 children of mean age 4·2 years showing close agreement for median values of total fat, saturated fat and total sugar expressed as a percentage of energy^(^ ^31^ ^)^. Two versions of the FFQ were used: one for children aged 3–11 years which had instructions for completion directed at the parent/guardian; and one for those aged 12–16 years which included six questions on coffee and alcoholic drinks and was designed for completion by the child with help from the parent/guardian. The instructions asked the children or parents/guardians to indicate the usual frequency of consumption of defined measures (e.g. ‘1 tablespoon’ or 1 piece’) of 140 (or 146) foods/drinks using nine response categories ranging from ‘rarely/never’ to ‘7 or more per day’. The FFQ aims to gather information on habitual consumption: as the instructions in the version for younger children were aimed at parents/guardians, the time frame suggested was ‘in the last 2–3 months’, but for the version for older children the phrasing was ‘in a typical week’. In 2006, as a check on validity of the FFQ in this study population, 429 randomly selected children and their parents/guardians where appropriate were invited to complete a single 24 h multiple-pass recall and a further 311 children and parents/guardians where appropriate were invited to keep a 4 d non-weighed food diary. Data from 350 children were obtained for both the FFQ and 24 h recall and from 153 children for both the FFQ and 4 d diet diary. For energy intake and NMES as a percentage of food energy, the values were on average 6 and 9 % higher, respectively, by the FFQ than by 24 h recall (both *P*=0·002) and 11 and 4 % higher by the FFQ than the diet diary (*P*<0·001 and *P*=0·056, respectively), although there was no significant difference between total fat or saturated fat as a percentage of food energy between the FFQ and the other two methods^(^ ^28^ ^)^. Trained field workers visited the home to collect data on sociodemographic characteristics and physical activity and inactivity, measure the child’s height and weight, and check and collect the FFQ. Questions on physical activity out of school, including walking, sport and exercise, active play, housework and gardening, and on time spent in front of a screen (television, computer or games console) out of school, were those used in the Scottish Health Survey^(^ ^5^ ^)^.

In 2006, 2498 children were invited to take part of whom 1700 (68 %) were interviewed and measured at home: FFQ data were obtained for 1512 (61 %) children with 1391 (56 %) FFQ used in the analysis. In 2010, 3048 children were invited to take part of whom 1906 (63 %) were interviewed and measured at home: FFQ data were obtained for 1816 (60 %) children with 1674 (55 %) FFQ used in the analysis. Reasons FFQ were omitted from the analysis included incomplete questionnaires (defined as more than ten missing responses) or extreme values for energy intake (defined as those above the 97·5th centile or below the 2·5th centile for energy intake within the age and sex group). A small number of children reached their 17th birthday between recruitment and measurement, so results are reported as including children aged up to 17 years.

BMI was calculated for children who had reliable measurements of both height and weight, with overweight (including obesity) and obesity defined as BMI ≥85th and ≥95th percentile, respectively, of UK reference data^(^ ^32^ ^)^. The FFQ was used to estimate habitual intake of thirty-three separate food groups of which seven groups were of particular interest in the present study: (i) sugar-sweetened soft drinks, (ii) confectionery and biscuits, (iii) cakes and pastries, all of which are indicator foods for assessment of progress in the Scottish Government Obesity Route Map^(^ ^33^ ^)^; (iv) crisps and savoury snacks, since higher-fat and -salt versions have not been available in schools in Scotland since 2008; and (v) fruit, (vi) fruit juice and (vii) vegetables, which are highlighted in the widely publicised ‘5 a day’ message. Intakes of all thirty-three food groups were used to estimate mean intakes of energy, NMES, total fat and saturated fat using the most recent version of the UK National Diet and Nutrition Survey nutrient databank available at the time of each survey^(^ ^34^ ^,^ ^35^ ^)^. Analysis by level of socio-economic deprivation used the child’s home postcode to obtain a score for the Scottish Index of Multiple Deprivation (SIMD). This is derived from thirty-eight indicators in seven domains (income, employment, housing, health, education, access to services and crime) using small area population census data, and is divided into five quintiles with quintile 1 being the most deprived and quintile 5 the least deprived^(^ ^36^ ^)^.

Statistical analysis was carried out using the statistical software packages Stata/SE 11.2 and IBM SPSS Statistics Version 22.0. Nutrient and food group variables that had a skewed distribution were transformed into a new variable as ln(±(old variable) – *k*), with *k* and the sign of the old variable chosen so that the skewness of the new variable was as close to zero as possible. Means and 95 % confidence intervals were converted back to the original scale for tabulation. Mean values were weighted to take account of sampling and non-response bias and to make the age and sex profiles of the weighted sample match those of the whole population using the survey commands in Stata or complex samples procedures in IBM SPSS Statistics. Differences between 2006 and 2010 were assessed by *t* test and linear associations between continuous variables and SIMD quintile were assessed by linear regression with SIMD as an ordinal variable. The significance of the difference in changes from 2006 to 2010 between the socio-economic groups was assessed by the interaction term in a general linear model with survey year and SIMD quintile as covariates.

## Results

Information on the intakes of the selected food groups in the two surveys is shown in Table 1. For all food groups apart from biscuits, cakes and pastries there were significant linear associations between the amounts reported to be consumed and SIMD quintile in both 2006 and 2010. There were small but statistically significant reductions in the amount of confectionery and crisps and savoury snacks consumed and an increase in the amount of vegetables consumed by all children between 2006 and 2010, with greater changes in mean intakes of sugar-sweetened beverages and vegetables in children in more deprived areas, but the interaction analysis found no significant change in the socio-economic gradients between the surveys.

**Table 1:** Selected food groups intakes of aged children 3–17 years classed as consumers of the food in 2006 and 2010 by quintile of the Scottish Index of Multiple Deprivation (SIMD)

	Survey	%	All children†		*P* for	SIMD quintile 1 (most deprived)		SIMD quintile 2		SIMD quintile 3		SIMD quintile 4		SIMD quintile 5 (least deprived)		*P* for linear	*P* for
Food group intake (g/d)	year	consumers*	Mean	95 % CI	difference‡	Mean	95 % CI	Mean	95 % CI	Mean	95 % CI	Mean	95 % CI	Mean	95 % CI	trend§	interaction‖
Confectionery	2006	98	22	21, 24	<0·001	29	26, 32	25	22, 28	21	18, 24	20	18, 23	19	17, 20	<0·001	0·793
	2010	99	19	18, 20		23	21, 26	20	18, 23	17	15, 19	18	16, 20	16	15, 18	<0·001	
Biscuits, cakes and pastries	2006	99	36	34, 37	0·277	36	32, 40	35	32, 39	34	31, 37	37	33, 41	36	33, 39	0·827	0·757
	2010	99	34	33, 36		32	29, 35	35	31, 38	34	31, 37	34	31, 38	36	33, 39	0·110	
Crisps and savoury snacks	2006	97	21	19, 22	<0·001	26	23, 30	24	21, 27	20	18, 23	19	16, 21	16	14, 19	<0·001	0·680
	2010	97	17	16, 18		23	21, 25	18	16, 20	16	15, 18	15	13, 16	14	13, 16	<0·001	
Sugar-sweetened soft drinks	2006	91	161	145, 179	0·056	234	204, 268	189	163, 218	145	124, 169	137	108, 173	123	104, 144	<0·001	0·927
	2010	91	139	129, 150		195	170, 224	164	141, 190	115	100, 133	119	103, 137	111	96, 127	<0·001	
Fruit juice and smoothies	2006	88	63	57, 68	0·209	41	36, 46	58	49, 67	59	51, 68	72	60, 86	87	76, 100	<0·001	0·307
	2010	89	57	53, 60		43	37, 50	54	47, 61	54	46, 62	59	53, 66	74	65, 84	<0·001	
Fruit (excl. fruit juice)	2006	98	132	125, 140	0·359	112	99, 127	138	124, 153	136	125, 148	130	118, 143	146	130, 163	0·007	0·426
	2010	98	128	122, 134		117	105, 130	127	115, 139	127	112, 143	137	125, 149	135	126, 145	0·009	
Vegetables (excl. potatoes)	2006	96	52	49, 56	0·002	43	39, 48	45	39, 52	55	49, 62	55	48, 63	65	57, 73	<0·001	0·482
	2010	95	59	56, 62		50	44, 56	53	48, 58	59	53, 67	68	61, 76	67	62, 72	<0·001	

* Children who consumed the food/drink at least once per month.

† Numbers of children in quintile 1–quintile 5: 253, 268, 259, 278 and 315 in 2006 and 314, 291, 305, 355 and 409 in 2010.

‡ Difference between 2006 and 2010.

§ Linear trend across SIMD quintiles.

‖ Interaction between survey year and SIMD.

Table 2 shows that between 2006 and 2010 there was a significant decrease in reported energy intake and in that derived from NMES in all socio-economic groups. There was no change in the percentage of food energy derived from total fat, although there was a significant reduction in the percentage of food energy derived from saturated fat. Table 2 also shows that energy intake and the percentage of food energy derived from NMES were significantly higher among children in more deprived areas in both surveys, although there were no socio-economic differences in intake in total fat or saturated fat as a percentage of food energy in either year. There was no significant change in socio-economic gradients in energy or nutrient intake between the two surveys.

**Table 2:** Energy, sugar and fat intakes, physical activity and inactivity, and overweight and obesity in children aged 3–17 years in 2006 and 2010 by quintile of the Scottish Index of Multiple Deprivation (SIMD)

	Survey	All children*		*P* for	SIMD quintile 1 (most deprived)		SIMD quintile 2		SIMD quintile 3		SIMD quintile 4		SIMD quintile 5 (least deprived)		*P* for linear	*P* for
Variable	year	Mean	95 % CI	difference†	Mean	95 % CI	Mean	95 % CI	Mean	95 % CI	Mean	95 % CI	Mean	95 % CI	trend‡	interaction§
Energy intake (MJ/d)	2006	7·68	7·53, 7·85	<0·001	7·98	7·66, 8·33	8·05	7·63, 8·49	7·44	7·20, 7·69	7·49	7·24, 7·76	7·51	7·26, 7·76	0·002	0·655
	2010	7·12	7·00, 7·25		7·58	7·32, 7·87	7·32	7·06, 7·59	6·87	6·61, 7·15	7·07	6·84, 7·32	6·78	6·58, 7·00	<0·001	
NMES (% food energy)	2006	17·4	17·0, 17·8	<0·001	18·4	17·6, 19·2	18·1	17·3, 18·9	16·8	16·1, 17·5	17·4	16·5, 18·3	16·3	15·7, 17·0	0·001	0·525
	2010	15·6	15·3, 16·0		16·7	15·8, 17·7	16·1	15·5, 16·7	15·0	14·5, 15·6	15·3	14·7, 15·9	15·2	14·7, 15·6	0·001	
Total fat (% food energy)	2006	32·9	32·7, 33·2	0·181	33·4	32·8, 34·1	32·8	32·2, 33·3	32·9	32·3, 33·5	32·7	32·3, 33·2	32·8	32·3, 33·3	0·162	0·662
	2010	32·7	32·5, 33·0		32·9	32·3, 33·4	32·8	32·3, 33·4	33·0	32·5, 33·4	32·7	32·3, 33·1	32·5	32·0, 32·9	0·219	
Saturated fat (% food energy)	2006	13·8	13·7, 14·0	<0·001	14·0	13·6, 14·3	13·8	13·6, 14·1	13·8	13·5, 14·1	13·8	14·6, 14·1	13·8	13·4, 14·2	0·542	0·365
	2010	13·2	13·1, 13·3		13·1	12·8, 13·4	13·1	12·8, 13·4	13·4	13·1, 13·7	13·2	13·0, 13·4	13·1	12·9, 13·3	0·970	
Physical activity out of school (h/week)	2006	17·5	16·1, 18·2	<0·001	17·3	15·6, 19·0	18·3	16·9, 19·9	17·7	16·1, 19·3	17·4	15·7, 19·2	15·8	14·5, 17·0	0·142	0·952
	2010	14·7	14·0, 15·4		15·0	13·8, 16·2	15·3	14·0, 16·7	14·2	12·8, 15·8	14·6	13·5, 15·7	13·3	12·3, 14·4	0·013	
Leisure-time screen use (h/d)	2006	1·8	1·7, 1·9	0·090	2·1	1·9, 2·2	1·9	1·8, 2·1	1·6	1·5, 1·8	1·8	1·6, 1·9	1·6	1·5, 1·7	<0·001	0·476
	2010	1·9	1·8, 2·0		2·2	2·1, 2·3	2·0	1·8, 2·1	1·8	1·7, 1·9	1·8	1·6, 1·9	1·7	1·6, 1·8	<0·001	
Overweight (incl. obese) (%)	2006	31·1	28·8, 33·4	0·901	31·7	26·8, 37·1	34·2	29·4, 39·5	35·6	29·3, 42·4	29·8	24·8, 35·3	25·0	21·6, 28·8	0·013	0·296
	2010	31·3	29·1, 33·6		37·5	32·9, 42·4	32·9	27·3, 39·0	29·3	24·8, 34·3	31·8	27·0, 37·0	25·2	21·1, 29·8	0·001	
Obese (%)	2006	16·6	14·5, 18·9	0·907	16·6	12·8, 21·2	17·8	14·4, 21·9	21·1	16·8, 21·2	16·3	12·5, 21·1	11·9	8·4, 16·7	0·091	0·065
	2010	16·8	15·1, 18·6		23·3	19·3, 27·8	17·7	13·6, 22·7	14·8	11·3, 19·2	16·3	12·5, 20·9	11·8	9·0, 15·2	<0·001	

NMES, non-milk extrinsic sugars.

* Numbers of children for energy, sugars and fat in quintile 1–quintile 5 as in Table 1. Numbers of children for activity in quintile 1–quintile 5: 375, 319, 312, 320 and 358 in 2006 and 370, 333, 353, 404 and 444 in 2010. Numbers of children for overweight and obesity in quintile 1–quintile 5: 350, 305, 303, 313 and 344 in 2006 and 345, 315, 343, 385 and 428 in 2010.

† Difference between 2006 and 2010.

‡ Linear trend across SIMD quintiles.

§ Interaction between survey year and SIMD.

In 2010, children living in the more deprived areas spent significantly more time in physical activity out of school but also significantly more leisure time in screen-based activities than children in less deprived areas. In children in all socio-economic groups the time spent in physical activity out of school decreased significantly between the two surveys while there was no significant change in leisure-time screen-based activity. There was also no significant difference in the socio-economic gradients in physical activity or leisure-time screen use between the two surveys.

The prevalence of overweight (including obesity) was 31 % and the prevalence of obesity was 17 % in both 2006 and 2010 (Table 2). In the 2006 survey the prevalence of overweight (including obesity) was 25 % in the least deprived quintile and 32 % in the most deprived quintile (prevalence ratio 1·28), while in 2010 the prevalence was also 25 % in the least deprived quintile but 38 % in the most deprived quintile (prevalence ratio 1·52). This pattern was driven by a difference in obesity prevalence, which was 12 % among children in the least deprived quintile in both years but among children in the most deprived quintile was 17 % in 2006 (prevalence ratio 1·41) and 23 % in 2010 (prevalence ratio 1·92). The changes in socio-economic gradients in overweight and obesity prevalence were not statistically significant.

## Discussion

The results from these two surveys suggest that there was some improvement in the diet of children in Scotland between 2006 and 2010, with an overall decrease in energy intake of 0·56 MJ/d (143 kcal/d) or about 7·3 %. The decreases in intakes of confectionery and crisps and savoury snacks were proportionately greater than the decrease in energy intake, while the 13 % increase in vegetable intake indicates a substantial change in diet over a relatively short time interval. At the time of the 2006 survey, nutritional guidance for school meals in Scotland^(^ ^13^ ^)^ had been implemented in primary schools and was in the process of being established in secondary schools, and between the two surveys legislation prohibiting sale of soft drinks, regular crisps and confectionery in all schools in Scotland came into force^(^ ^14^ ^,^ ^15^ ^)^. While these should have encouraged changes in diet in the directions seen, in the absence of data from a ‘control’ group of children it is not possible to attribute the changes in diet to the effects of these initiatives alone.

In both surveys there were socio-economic differences in the consumption of confectionery and sugar-sweetened soft drinks between children in the more and less deprived areas. However, the intake of NMES as a percentage of food energy was much higher than recommended levels among all children, with the lower intake of sugar-sweetened beverages among children in the less deprived areas partly compensated for by their higher intakes of fruit juice and smoothies.

In contrast to the socio-economic gradients in food and nutrient intakes, physical activity out of school was higher in children in the more-deprived areas, with the linear trend across quintiles being statistically significant in 2010. Unlike the improvement seen in diet, there was no evidence for a change in leisure-time screen use but there was a significant decline in physical activity out of school between the two surveys, which was of similar magnitude in all SIMD quintiles. A UK study of 10–11-year-olds in 2006–2007 which used accelerometery found that children in areas of greater socio-economic deprivation spent less time in sedentary behaviour (including screen-based and non-screen-based activities) after school and at weekends than those in other areas^(^ ^37^ ^)^, which contrasts with the pattern for screen-based activities reported here. The increase in the prevalence of obesity among children in the most deprived areas between the two surveys adds to the evidence for a widening socio-economic difference in child obesity prevalence in the UK^(^ ^10^ ^–^ ^12^ ^)^.

The present is the first quantitative analysis of socio-economic differences in children’s diet and activity behaviours over time in UK children. Strengths include the use of a whole population sampling frame, the large sample sizes and the use of the same methods for assessing diet and activity in the two surveys. Using weighting to adjust results for selection and non-response bias allows the data to reflect the true population mean more accurately. Another strength is the use of up-to-date food composition data for each survey, so that changes in energy and nutrient intakes observed include those due to changes in food composition. These include the changes due to reformulation and reduction in portion size of high-fat, high-sugar products as proposed in the saturated fat and energy reduction programme of the Food Standards Agency and Food Standards Agency Scotland announced in 2008–2010 and now the subject of pledges by a number of large manufacturers as part of the Public Health Responsibility Deal in England and Supporting Healthy Choices in Scotland^(^ ^38^ ^,^ ^39^ ^)^. When we used the 2006 food composition values to estimate nutrient intakes from the 2010 FFQ data to simulate no change in food composition^(^ ^29^ ^)^, the changes in energy and particularly NMES intakes between the surveys were attenuated, suggesting that changes in food composition have made an important contribution to the reduction in children’s NMES intake between the two surveys.

An important limitation of the present study is that all data on diet and activity were obtained by questionnaire responses from parents and/or children, which may have led to bias in the estimates. While there is no perfect objective measure of diet, in the 2006 survey the comparisons of the FFQ with a 24 h multiple-pass recall or a 4 d non-weighed food diary in random sub-samples found that differences in the estimated intakes of energy and NMES, total fat and saturated fat expressed as a percentage of food energy ranged from −1 to 11 % of the values by the reference methods^(^ ^28^ ^)^. For physical activity a comparison of the questionnaire used in the Health Survey for England with accelerometery in 8-year-olds found that the questions overestimated moderate-to-vigorous physical activity in children, so the true levels are probably lower than those reported here^(^ ^40^ ^)^. One further issue that could have influenced the comparison between years is a change in age and sex distribution of the study population. However, as the interval between the surveys was only 4 years and the age of the eligible children covered a 14-year age range, 70 % of children eligible in 2006 were also eligible in 2010 so population demographic change is unlikely to have contributed to the findings.

The present study results are broadly similar to analyses of data from the National Health and Nutrition Examination Surveys for US children, which show a reduction in energy intake and in added sugar content of the diet from 17·1 % of energy in 2003–2004 to 14·1 % of energy in 2009–2010 and a reduction in physical activity and an increase in sedentary activity among adolescents^(^ ^41^ ^,^ ^42^ ^)^. Other European countries show some different trends from those reported here; for example, in the Netherlands vegetable intake in 11-year-olds decreased between 2003 and 2009^(^ ^43^ ^)^ while among adolescents in Germany there was an increase in physical activity and a decrease in inactivity between 2002 and 2010^(^ ^44^ ^)^.

In summary, these surveys suggest that there was no convincing closing of the gap between socio-economic groups in terms of diet, physical activity or obesity between the two surveys; although the results for sugar-sweetened beverages and vegetables in children in the more deprived areas give some suggestion that the gap in consumption of these items may be beginning to narrow. Overall there were significant improvements in intakes of confectionery, crisps, savoury snacks and vegetables and in energy, NMES and saturated fat intakes, but NMES intake remains much higher than recommended levels in all children. Physical activity out of school, while higher among children in more deprived areas, fell significantly between the two surveys. Population-wide initiatives that can be effective in all socio-economic groups, such as changes in the marketing of energy-dense foods and drinks^(^ ^39^ ^,^ ^45^ ^)^ and legislation such as the recently proposed tax on sugar-sweetened soft drinks^(^ ^18^ ^)^, are needed to change the food environment for all children.

## Footnotes

* NMES includes all forms of free sugars plus 50 % of the sugars in dried, canned and stewed fruit.
